# Plasma BDNF and Cytokines Correlated with Protein Biomarkers for Bipolar II Disorder

**DOI:** 10.3390/jpm11121282

**Published:** 2021-12-02

**Authors:** Sheng-Yu Lee, Tzu-Yun Wang, Ru-Band Lu, Liang-Jen Wang, Cheng-Ho Chang, Yung-Chih Chiang, Chih-Chuan Pan, Kuo-Wang Tsai

**Affiliations:** 1 Department of Psychiatry, Kaohsiung Veterans General Hospital, Kaohsiung City 813414, Taiwan; sylee@vghks.gov.tw (S.-Y.L.); chhchang@vghks.gov.tw (C.-H.C.); ycchiang@vghks.gov.tw (Y.-C.C.); ccpan@vghks.gov.tw (C.-C.P.); 2 Department of Psychiatry, Faculty of Medicine, Kaohsiung Medical University, Kaohsiung City 80708, Taiwan; 3 Department of Psychiatry, National Cheng Kung University Hospital, College of Medicine, National Cheng Kung University, Tainan 70101, Taiwan; tzuyun0105@hotmail.com (T.-Y.W.); rblu_beijing@163.com (R.-B.L.); 4 Yanjiao Furen Hospital, Langfang 065201, Hebei, China; 5 Department of Child and Adolescent Psychiatry, Kaohsiung Chang Gung Memorial Hospital and Chang Gung University College of Medicine, Kaohsiung City 83301, Taiwan; anus78@cgmh.org.tw; 6 Department of Research, Taipei Tzu Chi Hospital, Buddhist Tzu Chi Medical Foundation, New Taipei City 231405, Taiwan

**Keywords:** bipolar II disorder, protein, plasma, BDNF, cytokines, correlation

## Abstract

We have previously identified five candidate proteins (matrix metallopeptidase 9 (MMP9), phenylalanyl-TRNA synthetase subunit beta (FARSB), peroxiredoxin 2 (PRDX2), carbonic anhydrase 1 (CA-1), and proprotein convertase subtilisin/kexin Type 9 (PCSK9)) as potential biomarkers for bipolar II disorder (BD-II). These candidate proteins have been associated with neuroprotective factors (BDNF) and inflammatory factors (cytokines, C-reactive protein (CRP), and tumor necrosis factor-α (TNF-α)). However, the correlations between these proteins with plasma BDNF and inflammatory factors remain unknown. We recruited a total of 185 patients with BD-II and 186 healthy controls. Plasma levels of candidate proteins, BDNF, cytokines (TNF-α, CRP, and interleukin-8 (IL-8)) were assessed from each participant. The correlations between levels of candidate proteins, BDNF, and cytokines were analyzed. In the BD-II group, we found that the level of FARSB was positively correlated with the BDNF level (r = 0.397, *p* < 0.001) and IL-8 (r = 0.320, *p* < 0.001). The CA-1 level positively correlated with IL-8 (r = 0.318, *p* < 0.001). In the control group, we found that the FARSB level positively correlated with the BDNF level (r = 0.648, *p* < 0.001). The CA-1 level positively correlated with TNF-α (r = 0.231, *p* = 0.002), while the MMP-9 level positively correlated with the CRP level (r = 0.227, *p* = 0.002). Our results may help in clarifying the underlying mechanism of these candidate proteins for BD-II.

## 1. Introduction

Bipolar II disorder (BD-II), one subtype of bipolar disorder, imposes a huge socioeconomic burden on the patient [1,2]. As BD-II is currently solely diagnosed through clinical presentation and assessment of the patient’s history, the diagnosis is frequently delayed or missed. The discovery of clinically applicable and reliable biomarkers for BD-II would therefore assist prompt diagnosis and lead to more effective treatment. Proteomics, the study of the identification and quantitative analysis of protein expression in an organism or system, has been proposed as a promising way to identify novel protein biomarkers for certain diseases [3]. We have previously identified candidate protein biomarkers for BD-II including PRDX2, CA-1, FARSB, MMP9, and PCSK9 [4]. The combination of these five candidate proteins was found to be able to predict the diagnosis of BD-II with good validity. However, the mechanisms underlying the associations between these proteins and BD-II pathogenesis remain unclear.

Dysregulation of cytokine-mediated inflammatory responses has gained increased attention as another pathogenesis of BD [5,6]. Strakowski et al. [7] reported that structural abnormalities in several brain areas including the prefrontal cortex, amygdala, and striatum lead to neuroinflammation and possible progression of brain atrophy; the inflammation has been associated with the exacerbation of clinical symptoms of BD. Levels of tumor necrosis factor (TNF)-α and C-reactive protein (CRP) were found to be increased during acute manic and depressive episodes and decreased after treatment [8,9]. In addition, elevated levels of IL-8 have also been associated with depressive states in BD. Cytokines have therefore been suggested as another diagnostic and staging biomarker for BD [10,11].

Brain-derived neurotrophic factor (BDNF), which is robustly expressed in the brain cortex and hippocampus, is an important protein for neuron development [12,13,14]. It has been suggested that peripheral BDNF levels reflect central BDNF levels as BDNF may cross the blood–brain barrier (BBB) freely [15,16]. Decreased peripheral BDNF levels have been correlated with worse depressive and manic symptoms [17] and later-staged BD [18,19]. Therefore, peripheral BDNF has been proposed as a potential biomarker of disease state and stage (progression) for BD [20].

Since in our previous study, most of the identified proteins (MMP9, PCSK9, FARSB, CA-1, and PRDX2) associated with BD-II were found to be related to the inflammatory process and changes in cytokines [21,22,23,24,25], it will be of interest to further elucidate the relations between these candidate proteins of BD-II with cytokines and BDNF.

In the current study, we thus aimed to clarify the correlations between the candidate plasma proteins of BD-II and cytokine and BDNF levels, in both patients with BD-II and normal controls. We hypothesized that there may be correlations between these proteins and inflammatory markers. In addition, we presumed to find differences in such correlations between patients with BD-II and controls, and expected our findings to provide novel insights into the underlying mechanisms of these earlier identified proteins associated with BD-II.

## 2. Methods

The research protocol for the current study was approved by the institutional review boards of Kaohsiung Veterans General Hospital (IRB No. VGHKS19-CT5-17) and National Cheng Kung University Hospital (IRB No. B-BR-108-046). The study was conducted at the psychiatry departments of both hospitals. Informed consent was obtained from each participant after the purpose of the study was explained. The study procedures abided by all relevant regulations and guidelines.

### 2.1. Patients

We recruited patients with BD-II, aged between 20 and 65 years old, from inpatient wards and outpatient psychiatric clinics at Kaohsiung Veterans General Hospital and National Cheng Kung University Hospital. All patients underwent a structural interview, the Chinese version of the Modified Schedule of Affective Disorders and Schizophrenia-Life Time (SADS-L) [26], conducted by research psychologists to confirm their clinical diagnosis. We adopted a 2-day minimum duration for the diagnosis of hypomanic episodes but not the 4-day duration defined in the Diagnostic and Statistical Manual of Mental Disorders, Fourth Edition Text Revision (DSM-IV-TR; American Psychiatric Association, 2000), because previous epidemiologic data [27,28] indicated that the 2-day minimum duration might be more appropriate in the community. The inclusion criterion for recruitment was a diagnosis of BD-II. The exclusion criteria were (a) any other major or minor mental illnesses besides BD-II such as psychotic disorders, personality disorders, substance use disorders, or organic mental disorders, and (b) any major medical or neurological disorders.

Healthy controls were recruited from the community and screened for psychiatric conditions using the SADS-L as a structural interview. The inclusion criteria for the controls were: (a) age between 20 and 65 years; (b) no diagnosis of any major or minor mental (illnesses including bipolar disorder, psychotic disorders, major or minor depressive disorders, substance use disorder, personality disorders, and organic mental disorders) and no family history of psychiatric disorder among their first-degree relatives; (c) no blood transfusions within the past month; and (d) no severe trauma within the past month.

### 2.2. Measures of Symptomatology

Clinical severity of mood was evaluated by research psychiatrists using the Hamilton Depression Rating Scale (HAMD) [29,30] for depressive symptoms and the Young Mania Rating Scale (YMRS) [31] for manic severity. 

The following categories of mood states according to the HAMD and YMRS rating scales were assessed at the clinical evaluation, without duration criteria: Depressive state (HAMD > 8 and YMRS < 8), hypomanic state (YMRS > 7 and HAMD < 8), mixed state (HAMD > 7 and YMRS > 7), and euthymic state (HAMD < 8 and YMRS < 8) [32].

### 2.3. Plasma Collection

Twenty-milliliter samples of whole blood were drawn from the antecubital vein of each patient and each control. The collected blood samples were placed in a test tube containing ethylenediaminetetraacetic acid (EDTA) (Greiner Bio-One Vacuette; Santa Cruz Biotechnology, Santa Cruz, CA, USA) and stored on ice for less than 30 min. The samples were then centrifuged at 3000× *g* for 15 min at 4 °C to isolate the plasma, which was then stored at −80 °C for further analysis. 

### 2.4. Evaluation of Concentrations of Candidate Proteins by ELISA

The plasma levels of MMP9, FARSB, PRDX2, CA-1, and PCSK9 proteins were assessed using commercial ELISA kits as follows: MMP9 (ARG80129, Arigo, Hsinchu, Taiwan), FARSB (EH8444, FineTest, Wuhan, China), PRDX2 (ARG82038, Arigo, Hsinchu, Taiwan), CA-1 (ARG82250, Arigo, Hsinchu, Taiwan), and PCSK9 (ARG81395, Arigo, Hsinchu, Taiwan). An enzyme-linked immunosorbent assay (ELISA) reader with a minimum detectable dose of 80 pg/mL (SpectraMax-M2; Molecular Devices, Sunnyvale, CA, USA) was used, and all samples were analyzed twice.

### 2.5. Plasma BDNF and Cytokine Assessment

Plasma BDNF levels were measured using a BDNF Kit (Quantikine Human BDNF kit; R&D Systems, Minneapolis, MN, USA) (www.RnDSystems.com) and an ELISA reader with a minimum detectable dose of 80 pg/mL (SpectraMax-M2; Molecular Devices, Sunnyvale, CA, USA). All samples were analyzed twice. The cytokine ELISA kits used were also purchased from R&D systems and included a Human CRP Quantikine ELISA Kit, a Human TNF-alpha Quantikine ELISA Kit, and a Human IL-8 Quantikine ELISA Kit for the assessment of plasma levels of CRP, TNF-α, and IL-8, respectively.

### 2.6. Statistical Analysis

Since all protein, BDNF, and cytokine level data were skewed to the right and did not follow normal distributions, we applied log10 transformations and used parametric tests for analysis. Differences in correlations of clinical variables with plasma protein, BDNF, and cytokine levels between patients and controls were analyzed using *t*-tests and chi-square tests. Correlations between plasma protein levels and levels of BDNF and cytokines were analyzed using Pearson’s correlation. On the other hand, additionally non-parametric tests (the Mann–Whitney U test and Spearman’s correlation) were analyzed for non-transformed data. The statistical software SPSS v25.0 (IBM Corp., Armonk, NY, USA) was used for all statistical analyses.

## 3. Results

A total of 185 patients with BD-II and 186 controls were recruited; their clinical characteristics are listed in Table 1 All recruited patients were diagnosed with BD-II for the first time and had not previously received treatment for BD. Of all patients, 22 were in a depressive state, 12 were in a hypomanic state, 143 were in a mixed state, and eight were in a euthymic state. About 89.7% of our patients were recruited from one of the outpatient clinics, while only 10.2% were recruited from an inpatient ward (Table 1). After recruitment and blood sample collection, the patients were administered medication according to their clinical condition (lorazepam, 70.6% of patients; risperidone, 50.9%; valproate, 88.2%).

The blood samples of all participants were collected before noon between 9 am and 12 pm. We found significantly increased plasma proteins including PRDX2, CA-1, FARSB, MMP9, and PCSK9 and increased plasma levels of BDNF, CRP, and IL-8 in patients with BD-II compared to the controls (Table 1). However, there was no difference in the plasma levels of TNF-α between the patient and the control group. 

The correlations between plasma levels of proteins and levels of cytokines and BDNF of the patients are described in Table 2, and correlations for the control group are described in Table 3. To correct for multiple comparisons, we adjusted all *p* values by dividing the usual (0.05) significance level by 20 (4 × 5), thus accepting *p* < 0.0025 as significant. In the BD-II group, we found that levels of FARSB positively correlated with BDNF (r = 0.397, *p* < 0.001) and IL-8 levels (r = 0.320, *p* < 0.001). CA-1 levels positively correlated with IL-8 (r = 0.318, *p* < 0.001) (Table 2). After controlling for age, gender, and symptom severity using partial correlation, the positive correlations found in the BD-II group remained (data no shown). In the control group, we found that FARSB levels were positively correlated with BDNF levels (r = 0.648, *p* < 0.001). Furthermore, CA-1 levels positively correlated with TNF-α levels (r = 0.231, *p* = 0.002), while MMP-9 levels positively correlated with CRP (r = 0.227, *p* = 0.002) (Table 3). 

To account for the non-normal distribution of our data, we re-ran most of our analyses using the Mann–Whitney U test and Spearman’s correlation. Most findings remained positive using these non-parametric tests (Table 1, Table 2 and Table 3).

We then reanalyzed the observed correlations by dividing patients into the different mood states described above (the results are presented in Table 4). Due to the small numbers of patients in the depressive, hypomanic, and euthymic groups, our major findings were not observed in these but only in the mixed state group. 

Finally, we conducted a power analysis using G-Power 3.1.9.2 [33,34], with effect-size conventions determined according to Buchner et al. [33]. Our estimation yielded a power of approximately 0.60 to detect a small effect and a power of 0.99–1.0 to detect medium and large effects using the independent t-test for the BD-II and control groups (n = 371); small effect size was set at 0.2, medium effect size at 0.5, large effect size at 0.8, and alpha at 0.05 [33]. For the correlation analysis, our estimation for the two groups (n = 185 and n = 186) yielded a power of approximately 0.27 to detect a small effect, and a power of 0.99–1.0 to detect medium and large effects, with a small effect size of 0.1, a medium effect size of 03, a large effect size of 0.5, and alpha set at 0.05 [33].

## 4. Discussion

In the current study, we found significant correlations between plasma levels of candidate proteins of BD-II and cytokine and BDNF levels; however, the observed correlations differed between patients with BD-II and the controls. By correlating these candidate proteins with inflammatory markers, our study provides initial evidence to explain how these proteins participate in the mechanisms underlying BD-II. 

### 4.1. FARSB Protein and BDNF

We found significant increases in plasma levels of the FARSB protein and BDNF in patients with BD-II compared to the controls. In addition, we found a significantly positive correlation between levels of FARSB and BDNF in both patients and controls. A previous study reported decreased peripheral BDNF in BD in the later stage [19]; however, one recent study found that BDNF levels were increased in early-staged BD [35]. The study by Petersen et al. [35] suggested that increased BDNF in patients with BD in the early stage of the illness compared to controls may be explained by (1) younger age of the study group as well as (2) in-time diagnosis and in-time treatment intervention. Another meta-analysis comprised of 3798 participants found that BDNF levels increased with illness duration of BD [36]. In the current study, since all patients were diagnosed with BD-II for the first time, it is possible that the elevated BDNF levels stem from a compensatory or protective effect that occurs in the early stage of BD-II, as shown in the premorbid phase in individuals with a familial risk of BD [37]. 

FARSB is an enzyme belonging to the aminoacyl-tRNA synthetase family. Mutations in the FARSB gene have been associated with aminoacyl-tRNA synthetase-related diseases including cerebral aneurysms, brain calcifications, cirrhosis, and interstitial lung disease [22]. FARSB expression was found to be increased in dorsal horn neurons after peripheral nerve injury; FARSB has therefore been proposed as a neurotransmitter that may relay unusual sensory signals after peripheral nerve damage [23]. In the current study, we found significant correlations between BDNF and FARSB levels in both patients with BD-II and healthy controls that have not been reported in previous studies. We propose that if FARSB functions as a neurotransmitter associated with neuronal injury, BDNF may increase and exert a compensatory effect in response to the increase in FASRB. Our findings also showed significant correlations between levels of IL-8 and FARSB in patients with BD-II. Serum IL-8 levels have been found to be increased in patients with BD-II depression, and an increase in IL-8 has also been inversely correlated with functional connectivity in the right precentral gyrus [38]. The positive correlation between FARSB and IL-8 levels seems to support the notion that inflammation plays an important role in the pathogenesis of BD-II.

### 4.2. CA-1 and IL-8 Levels

We also found a significant increase in CA-1 and IL-8 levels in patients with BD-II compared to the controls; however, a significantly positive correlation between CA-1 and IL-8 was found only in the BD-II group. CA-1 is an enzyme primarily found in red blood cells, neutrophils, and the colonic epithelium [39], which catalyzes the interconversion between water and carbon dioxide [40]. Increased expression of CA-1 mediated calcification and cytokines has been found in atherosclerosis in a mouse model [41]. In another animal model, subdural infusion of CA-1 induced cerebral vascular permeability, which might contribute to neurovascular edema [42]. One previous study reported downregulation of plasma CA-1 in patients with BD during depressive episodes [43]; however, another study reported an increase in CA-1 levels in the brains of patients with major depressive disorder [44]. It has also been found that treating patients with BD in a depressive episode with carbonic anhydrase inhibitors may significantly improve clinical symptoms [45]. The correlation between CA-1 and IL-8 in BD observed in the current study indicates that CA-1 is involved in the patho-mechanism of BD-II through the cytokine system. 

### 4.3. Other Correlations

We found significant correlations of CA-1 with TNF-α and MMP9 with CRP, respectively, but only in our control sample. By increasing the permeability of the BBB, MMP9 increases the entry of cytokines into the central nervous system [24]. On the other hand, inflammatory cytokines are potent activators of the matrix metalloproteinase (MMP). In normal controls, MMP-9 levels have been associated with cardiovascular risk and psychosocial factors [46,47]. Previous studies have reported a significant positive correlation between serum levels of MMP-9 and CRP in patients with colorectal carcinogenesis [48] and in a middle-aged normal population [46], but such correlations have not been reported for BD. Our study revealed the same positive correlation in normal controls that was reported by Garvin et al. [46]; however, whether these two proteins can be used as biomarkers and predictors for other inflammatory diseases still requires further investigation.

### 4.4. Limitations

Our study has the following limitations that should be considered in the evaluation of our findings. First, due to the cross-sectional study design, we were unable to relate the changes in each candidate protein to the changes in BDNF and cytokines. Future longitudinal study will be needed to monitor changes in candidate proteins and changes in BDNF and cytokine levels during the progression of illness to elucidate whether the patho-mechanisms of these candidate proteins for BD-II are related to the neuroprotective and inflammatory pathways. Second, in the assessment of cytokine levels, it would have been useful to know whether patients and controls were under treatment with anti-inflammatory drugs. Since we excluded patients with any significant medical or neurological illnesses, we can suppose that the selected patients were not taking any long-term anti-inflammatory medication. However, we did not specifically control for sporadic anti-inflammatory drug use. In addition, we did not control for some common metabolic comorbidities such as diabetes and hypertension in our control sample, which may also have influenced the observed levels of cytokines. Future studies need to pay more attention to such details. Third, fasting times before blood sampling, which may also affect levels of cytokines, were not controlled for. Fourth, some protein as well as BDNF levels may change with age, and the significant differences in age and gender between patients and controls in the current sample may therefore have confounded the validity of our results. Future studies recruiting age- and gender-matched case and control groups may be warranted. 

### 4.5. Conclusions

We report positive correlations between plasma levels of the FARSB protein and BDNF and IL-8, and of CA-1 levels and IL-8 in patients with BD-II. In addition, we found positive correlations between plasma levels of the FARSB protein with those of BDNF, between levels of CA-1 and TNF-α, and between levels of MMP9 and CRP in a normal control group. Our positive findings suggest that inflammatory and neurodegenerative mechanisms are underlying the pathogenesis of these proteins in BD-II. Our findings also provide biological support for the diagnostic model we built using the combination of the five proteins investigated here, which may aid with the prompt identification of BD-II.

## Figures and Tables

**Table 1:** Clinical and demographic data of patients with bipolar II disorder (BD-II) and healthy controls.

	Training Cohort				
	BD-II	Healthy Controls	Χ^2^ or t	*p* Value ^a^	*p* Value ^b^
***n***	185	186			
**Age (mean, SD)**	36.2 ± 12.5	32.2 ± 8.9	3.63	<0.001 **	
**Gender (M/F)**	72/113	105/81	11.4	<0.001 **	
**Education (year)**	13.9 ± 2.5	15.5 ± 1.9	−6.2	<0.001 **	
**Onset age (year)**	15.2 ± 5.3	N/A			
**HAMD**	13.3 ± 4.0	N/A			
**YMRS**	12.4 ± 3.2	N/A			
**OPD/inpatient (*n*, %)**	166/19 89.8%/10.2%				
**Mood state (depressive/hypomanic/mixed/euthymic) (*n*, %)**	22/12/143/811.8/6.5/77.3/4.3				
**PRDX2 (ng/mL)**	12.64 ± 13.68	8.79 ± 8.82	3.03	0.003 *	0.027
**CA-1 (ng/mL)**	2562.0 ± 1937.3	1886.8 ± 1430.3	3.77	<0.001 **	<0.001 **
**FARSB (ng/mL)**	22.2 ± 12.1	16.3 ± 9.8	5.16	<0.001 **	<0.001 **
**MMP9 (ng/mL)**	104.9 ± 61.9	66.1 ± 44.2	6.87	<0.001 **	<0.001 **
**PCSK9 (ng/mL)**	324.8 ± 170.7	199.6 ± 87.2	8.77	<0.001 **	<0.001 **
**TNF-α (pg/mL)**	1.70 ± 2.05	1.60 ± 1.75	0.52	0.606	0.804
**CRP (ng/mL)**	2100.8 ± 1444.4	1593.0 ± 1340.9	3.47	0.001 **	<0.001 **
**IL8 (pg/mL)**	5.47 ± 4.27	2.42 ± 3.81	7.22	<0.001 **	<0.001 **
**BDNF (pg/mL)**	23862.6 ± 8894.8	17,534.0 ± 9426.8	6.59	<0.001 **	<0.001 **

HAMD: Hamilton depression rating scale; YMRS: Young manic rating scale. MMP9: Matrix metallopeptidase 9; FARSB: Phenylalanyl-TRNA synthetase subunit beta; PRDX2: Peroxiredoxin 2; CA-1: Carbonic anhydrase 1; PCSK9: Proprotein. Convertase subtilisin/kexin type 9. BDNF: Brain derived neuroprotective factors; CRP: C-reactive protein; TNF-α: Tumor necrosis factor-α; IL-8: Interleukin-8. N/A: Not available; SD: Standard deviation. We used the t-test for continuous variables and chi-square test for categorical variables. * *p* < 0.05; ** *p* < 0.01. ^a^ Calculated using transformed data (Log10 transformation of original data for normal distributions), ^b^ Calculated using non-parametric tests (Mann–Whitney U test).

**Table 2:** Correlation between level of protein levels and cytokine and BDNF in BD-II.

**(a) Log-transformed data using Pearson’s correlation.**								
	**BDNF**		**TNFα**		**CRP**		**IL-8**	
	**r**	***p***	**r**	***p***	**r**	***p***	**r**	***p***
**PRDX2 ^a^**	0.017	0.833	0.088	0.291	−0.147	0.067	0.018	0.831
**CA-1 ^a^**	0.062	0.425	0.142	0.076	−0.026	0.739	0.318	<0.001 **
**FARSB ^a^**	0.397	<0.001 **	−0.163	0.037	0.037	0.625	0.320	<0.001 **
**MMP9 ^a^**	0.015	0.843	−0.052	0.511	0.126	0.100	0.007	0.929
**PCSK9 ^a^**	0.177	0.018	0.099	0.207	0.131	0.086	−0.207	0.008
**(b) Original data using Spearman’s correlation.**								
	**BDNF**		**TNFα**		**CRP**		**IL-8**	
	**rho**	***p***	**rho**	***p***	**rho**	***p***	**rho**	***p***
**PRDX2**	0.039	0.623	0.042	0.595	−0.172	0.031	0.000	0.997
**CA-1**	0.036	0.645	0.032	0.674	−0.075	0.331	0.227	0.003
**FARSB**	0.362	<0.001 **	−0.004	0.957	0.044	0.561	0.392	<0.001 **
**MMP9**	−0.032	0.673	−0.022	0.769	0.089	0.241	0.041	0.587
**PCSK9**	0.245	0.001 *	0.042	0.579	0.145	0.056	−0.167	0.026

(a) r: Pearson’s correlation coefficient, ** *p* < 0.001, ^a^ Calculated using transformed data (Log10 transformation of original data for normal distribution). (b) Calculated using original data; rho: Spearman’s rho coefficient, * *p* < 0.0025; ** *p* < 0.001, MMP9: Matrix metallopeptidase 9; FARSB: Phenylalanyl-TRNA synthetase subunit beta; PRDX2: Peroxiredoxin 2; CA-1: Carbonic anhydrase 1; PCSK9: Proprotein convertase subtilisin/kexin type 9. BDNF: Brain derived neuroprotective factors; CRP: C-reactive protein; TNF-α: Tumor necrosis factor-α; IL-8: Interleukin-8.

**Table 3:** Correlation between level of protein levels and cytokine and BDNF in the controls.

**(a) Log-transformed data using Pearson’s correlation.**								
	**BDNF**		**TNFα**		**CRP**		**IL-8**	
	**r**	***p***	**r**	***p***	**r**	***p***	**r**	***p***
**PRDX2 ^a^**	−0.065	0.404	0.178	0.032	−0.130	0.094	0.006	0.950
**CA-1 ^a^**	−0.184	0.012	0.239	0.002 *	−0.034	0.647	0.109	0.275
**FARSB ^a^**	0.648	<0.001 **	−0.209	0.008	0.188	0.010	0.140	0.161
**MMP9 ^a^**	−0.010	0.895	0.103	0.196	0.227	0.002 *	0.057	0.566
**PCSK9 ^a^**	0.152	0.038	−0.041	0.611	−0.010	0.888	0.121	0.226
**(b) Original data using Spearman’s correlation.**								
	**BDNF**		**TNFα**		**CRP**		**IL-8**	
	**rho**	***p***	**rho**	***p***	**rho**	***p***	**rho**	***p***
**PRDX2**	0.121	0.119	0.072	0.344	−0.111	0.152	−0.036	0.644
**CA-1**	−0.194	0.008	0.240	0.001 *	−0.095	0.196	0.174	0.017
**FARSB**	0.688	<0.001 **	−0.132	0.072	0.272	<0.001 **	0.234	0.001 *
**MMP9**	−0.016	0.831	0.121	0.100	0.234	0.001 *	0.279	<0.001 **
**PCSK9**	0.188	0.010	0.035	0.637	0.018	0.806	0.152	0.038

(a) r: Pearson’s coefficient, * *p* < 0.0025; ** *p* < 0.001, ^a^ Calculated using transformed data (Log10 transformation of original data for normal distribution). (b) Calculated using original data; rho: Spearman’s rho coefficient, * *p* < 0.0025; ** *p* < 0.001, MMP9: Matrix metallopeptidase 9; FARSB: Phenylalanyl-TRNA synthetase subunit beta; PRDX2: Peroxiredoxin 2; CA-1: Carbonic anhydrase 1; PCSK9: Proprotein convertase subtilisin/kexin type 9. BDNF: Brain derived neuroprotective factors; CRP: C-reactive protein; TNF-α: Tumor necrosis factor-α; IL-8: Interleukin-8.

**Table 4:** Correlation between level of protein levels and cytokine and BDNF in BD-II divided by different mood state.

**(a) Depressive state (*n* = 22).**								
	**BDNF**		**TNFα**		**CRP**		**IL-8**	
	**rho**	***p***	**rho**	***p***	**rho**	***p***	**rho**	***p***
**PRDX2**	0.059	0.829	0.072	0.791	−0.362	0.169	0.075	0.782
**CA-1**	0.159	0.541	−0.444	0.074	−0.140	0.593	0.369	0.145
**FARSB**	0.096	0.705	−0.045	0.858	0.234	0.349	0.199	0.428
**MMP9**	−0.069	0.785	−0.366	0.136	−0.104	0.681	0.051	0.842
**PCSK9**	0.251	0.316	0.142	0.573	0.172	0.494	0.154	0.542
**(b) Hypomanic state (*n* = 12).**								
	**BDNF**		**TNFα**		**CRP**		**IL-8**	
	**rho**	***p***	**rho**	***p***	**rho**	***p***	**rho**	***p***
**PRDX2**	−0.527	0.096	0.000	1.000	−0.355	0.285	−0.064	0.853
**CA-1**	0.070	0.829	−0.434	0.159	−0.224	0.484	0.413	0.183
**FARSB**	−0.343	0.276	−0.210	0.513	−0.329	0.297	0.182	0.572
**MMP9**	−0.524	0.080	−0.105	0.746	−0.622	0.031	0.063	0.846
**PCSK9**	−0.399	0.199	0.273	0.391	0.392	0.208	−0.119	0.713
**(c) Mixed state (*n* = 143).**								
	**BDNF**		**TNFα**		**CRP**		**IL-8**	
	**rho**	***p***	**rho**	***p***	**rho**	***p***	**rho**	***p***
**PRDX2**	0.091	0.315	0.042	0.643	−0.131	0.154	0.044	0.633
**CA-1**	0.001	0.988	0.143	0.106	−0.040	0.652	0.183	0.039
**FARSB**	0.486	<0.001 **	−0.013	0.881	0.005	0.951	0.441	<0.001 **
**MMP9**	−0.033	0.705	0.033	0.706	0.167	0.058	0.052	0.550
**PCSK9**	0.321	<0.001 **	−0.006	0.948	0.138	0.118	−0.190	0.029
**(d) Euthymic state (*n* = 8).**								
	**BDNF**		**TNFα**		**CRP**		**IL-8**	
	**rho**	***p***	**rho**	***p***	**rho**	***p***	**rho**	***p***
**PRDX2**	0.300	0.624	−0.300	0.624	−0.600	0.400	0.100	0.873
**CA-1**	0.314	0.544	−0.429	0.397	0.200	0.704	0.371	0.468
**FARSB**	0.405	0.320	0.381	0.352	0.429	0.337	0.762	0.028
**MMP9**	0.095	0.823	−0.548	0.160	0.500	0.253	0.238	0.570
**PCSK9**	−0.405	0.320	0.024	0.955	−0.036	0.939	−0.619	0.102

Calculated using original data; rho: Spearman’s rho coefficient, ** *p* < 0.001, MMP9: Matrix metallopeptidase 9; FARSB: Phenylalanyl-TRNA synthetase subunit beta; PRDX2: Peroxiredoxin 2; CA-1: Carbonic anhydrase 1; PCSK9: Proprotein convertase subtilisin/kexin type 9. BDNF: Brain derived neuroprotective factors; CRP: C-reactive protein; TNFα: Tumor necrosis factor-α; IL-8: Interleukin-8.

## Data Availability

The data from this study are available from the corresponding author upon reasonable request.

## References

[1] Ghaemi S.N., Boiman E., Goodwin F.K. (2000). Insight and outcome in bipolar, unipolar, and anxiety disorders. Compr. Psychiatry.

[2] MacQueen G.M., Young L.T. (2001). Bipolar II Disorder: Symptoms, Course, and Response to Treatment. Psychiatr. Serv..

[3] Taurines R., Dudley E., Grassl J., Warnke A., Gerlach M., Coogan A., Thome J. (2010). Proteomic research in psychiatry. J. Psychopharmacol..

[4] Lee S.-Y., Wang T.-Y., Lu R.-B., Wang L.-J., Li S.-C., Tu C.-Y., Chang C.-H., Chiang Y.-C., Tsai K.-W. (2021). Identification of potential plasma protein biomarkers for bipolar II disorder: A preliminary/exploratory study. Sci. Rep..

[5] Goldstein B.I., Kemp D.E., Soczynska J., McIntyre R.S. (2009). Inflammation and the Phenomenology, Pathophysiology, Comorbidity, and Treatment of Bipolar Disorder: A systematic review of the literature. J. Clin. Psychiatry.

[6] Rosenblat J.D., Kakar R., Berk M., Kessing L., Vinberg M., Baune B.T., Mansur R.B., Brietzke E., Goldstein B.I., McIntyre R.S. (2016). Anti-inflammatory agents in the treatment of bipolar depression: A systematic review and meta-analysis. Bipolar Disord..

[7] Strakowski S.M., DelBello M.P., Adler C., Cecil K., Sax K.W. (2000). Neuroimaging in bipolar disorder. Bipolar Disord..

[8] Fernandes B.S., Steiner J., Molendijk M.L., Dodd S., Nardin P., Gonçalves C.-A., Jacka F., Köhler C.A., Karmakar C., Carvalho A.F. (2016). C-reactive protein concentrations across the mood spectrum in bipolar disorder: A systematic review and meta-analysis. Lancet Psychiatry.

[9] Goldsmith D.R., Rapaport M.H., Miller B.J. (2016). A meta-analysis of blood cytokine network alterations in psychiatric patients: Comparisons between schizophrenia, bipolar disorder and depression. Mol. Psychiatry.

[10] Goldstein B.I., Lotrich F., Axelson D.A., Gill M.K., Hower H., Goldstein T.R., Fan J., Yen S., Diler R., Dickstein D. (2015). Inflammatory markers among adolescents and young adults with bipolar spectrum disorders. J. Clin. Psychiatry.

[11] Jacoby A.S., Munkholm K., Vinberg M., Pedersen B.K., Kessing L.V. (2016). Cytokines, brain-derived neurotrophic factor and C-reactive protein in bipolar I disorder—Results from a prospective study. J. Affect. Disord..

[12] Monteggia L.M., Barrot M., Powell C., Berton O., Galanis V., Gemelli T., Meuth S., Nagy A., Greene R., Nestler E.J. (2004). Essential role of brain-derived neurotrophic factor in adult hippocampal function. Proc. Natl. Acad. Sci. USA.

[13] Cotman C.W., Berchtold N.C. (2002). Exercise: A behavioral intervention to enhance brain health and plasticity. Trends Neurosci..

[14] Hofer M., Pagliusi S.R., Hohn A., Leibrock J., Barde Y. (1990). Regional distribution of brain-derived neurotrophic factor mRNA in the adult mouse brain. EMBO J..

[15] Pan W., Banks W.A., Fasold M.B., Bluth J., Kastin A.J. (1998). Transport of brain-derived neurotrophic factor across the blood–brain barrier. Neuropharmacology.

[16] Klein A.B., Williamson R., Santini M.A., Clemmensen C., Ettrup A., Rios M., Knudsen G.M., Aznar S. (2010). Blood BDNF concentrations reflect brain-tissue BDNF levels across species. Int. J. Neuropsychopharmacol..

[17] Molendijk M., Spinhoven P., Polak M., Bus B.A.A., Penninx B.W.J.H., Elzinga B.M. (2013). Serum BDNF concentrations as peripheral manifestations of depression: Evidence from a systematic review and meta-analyses on 179 associations (N = 9484). Mol. Psychiatry.

[18] Kauer-Sant’Anna M., Kapczinski F., Andreazza A.C., Bond D.J., Lam R.W., Young L.T., Yatham L.N. (2009). Brain-derived neurotrophic factor and inflammatory markers in patients with early- vs. late-stage bipolar disorder. Int. J. Neuropsychopharmacol..

[19] Fernandes B.S., Molendijk M.L., Kohler C.A., Soares J.C., Leite C.M.G.S., Machado-Vieira R., Ribeiro T.L., Silva J.C., Sales P.M.G., Quevedo J. (2015). Peripheral brain-derived neurotrophic factor (BDNF) as a biomarker in bipolar disorder: A meta-analysis of 52 studies. BMC Med..

[20] Fernandes B.S., Gama C.S., Ceresér K.M., Yatham L.N., Fries G.R., Colpo G.D., Lucena D., Kunz M., Gomes F.A., Kapczinski F. (2011). Brain-derived neurotrophic factor as a state-marker of mood episodes in bipolar disorders: A systematic review and meta-regression analysis. J. Psychiatr. Res..

[21] Robinson C.E., Kottapalli V., D’Astice M., Fields J.Z., Winship D., Keshavarzian A. (1997). Regulation of neutrophils in ulcerative colitis by colonic factors: A possible mechanism of neutrophil activation and tissue damage. J. Lab. Clin. Med..

[22] Xu Z., Lo W.-S., Beck D.B., Schuch L.A., Oláhová M., Kopajtich R., Chong Y.E., Alston C., Seidl E., Zhai L. (2018). Bi-allelic Mutations in Phe-tRNA Synthetase Associated with a Multi-system Pulmonary Disease Support Non-translational Function. Am. J. Hum. Genet..

[23] Jung J., Jeong N.Y., Park B.S., Yeo S.G. (2015). A novel therapeutic target for peripheral nerve injury-related diseases: Aminoacyl-tRNA synthetases. Neural Regen. Res..

[24] Turner R.J., Sharp F.R. (2016). Implications of MMP9 for Blood Brain Barrier Disruption and Hemorrhagic Transformation Following Ischemic Stroke. Front. Cell. Neurosci..

[25] Momtazi A.A., Sabouri-Rad S., Gotto A.M., Pirro M., Banach M., Awan Z., Barreto G.E., Sahebkar A. (2019). PCSK9 and inflammation: A review of experimental and clinical evidence. Eur. Heart J.-Cardiovasc. Pharmacother..

[26] Endicott J. (1978). A Diagnostic Interview: The schedule for affective disorders and schizophrenia. Arch. Gen. Psychiatry.

[27] Benazzi F. (2007). Testing predictors of bipolar-II disorder with a 2-day minimum duration of hypomania. Psychiatry Res..

[28] Angst J., Gamma A., Benazzi F., Ajdacic-Gross V., Eich D., Rössler W. (2003). Toward a re-definition of subthreshold bipolarity: Epidemiology and proposed criteria for bipolar-II, minor bipolar disorders and hypomania. J. Affect. Disord..

[29] Hamilton M. (1960). A RATING SCALE FOR DEPRESSION. J. Neurol. Neurosurg. Psychiatry.

[30] Hamilton M. (1967). Development of a Rating Scale for Primary Depressive Illness. Br. J. Soc. Clin. Psychol..

[31] Young R.C., Biggs J.T., Ziegler V.E., Meyer D.A. (1978). A Rating Scale for Mania: Reliability, Validity and Sensitivity. Br. J. Psychiatry.

[32] Zimmerman M., Martinez J.H., Young D., Chelminski I., Dalrymple K. (2013). Severity classification on the Hamilton depression rating scale. J. Affect. Disord..

[33] Buchner A., Faul F., Erdfelder E. (1996). G-power: A Priori, Post Hoc, and Compromise Power Analyses for the Macintosh.

[34] Faul F., Erdfelder E., Buchner A., Lang A.-G. (2009). Statistical power analyses using G*Power 3.1: Tests for correlation and regression analyses. Behav. Res. Methods.

[35] Petersen N.A., Nielsen M.Ø., Coello K., Stanislaus S., Melbye S., Kjærstad H.L., Sletved K.S.O., McIntyre R.S., Frikke-Smith R., Vinberg M. (2021). Brain-derived neurotrophic factor levels in newly diagnosed patients with bipolar disorder, their unaffected first-degree relatives and healthy controls. BJPsych Open.

[36] Munkholm K., Vinberg M., Kessing L. (2015). Peripheral blood brain-derived neurotrophic factor in bipolar disorder: A comprehensive systematic review and meta-analysis. Mol. Psychiatry.

[37] Knorr U., Søndergaard M.H.G., Koefoed P., Jørgensen A., Faurholt-Jepsen M., Vinberg M., Kessing L. (2017). Increased blood BDNF in healthy individuals with a family history of depression. Psychiatry Res..

[38] Tang G., Chen P., Chen G., Zhong S., Gong J., Zhong H., Ye T., Chen F., Wang J., Luo Z. (2021). Inflammation is correlated with abnormal functional connectivity in unmedicated bipolar depression: An independent component analysis study of resting-state fMRI. Psychol. Med..

[39] Torella D., Ellison G.M., Torella M., Vicinanza C., Aquila I., Iaconetti C., Scalise M., Marino F., Henning B.J., Lewis F.C. (2014). Carbonic Anhydrase Activation Is Associated with Worsened Pathological Remodeling in Human Ischemic Diabetic Cardiomyopathy. J. Am. Heart Assoc..

[40] Supuran C.T. (2008). Carbonic Anhydrases An Overview. Curr. Pharm. Des..

[41] Yuan L., Wang M., Liu T., Lei Y., Miao Q., Li Q., Wang H., Zhang G., Hou Y., Chang X. (2019). Carbonic Anhydrase 1-Mediated Calcification Is Associated with Atherosclerosis, and Methazolamide Alleviates Its Pathogenesis. Front. Pharmacol..

[42] Gao B.-B., Clermont A., Rook S., Fonda S.J., Srinivasan V.J., Wojtkowski M., Fujimoto J.G., Avery R.L., Arrigg P.G., Bursell S.-E. (2007). Extracellular carbonic anhydrase mediates hemorrhagic retinal and cerebral vascular permeability through prekallikrein activation. Nat. Med..

[43] Song Y., Wu B., Yang Y., Chen J., Zhang L., Zhang Z., Shi H., Huang C., Pan J., Xie P. (2015). Specific alterations in plasma proteins during depressed, manic, and euthymic states of bipolar disorder. Braz. J. Med. Biol. Res..

[44] Johnston-Wilson N.L., Sims C.D., Hofmann J.-P., Anderson L., Shore A.D., Torrey E.F., Yolken R.H., The Stanley Neuropathology Consortium (2000). Disease-specific alterations in frontal cortex brain proteins in schizophrenia, bipolar disorder, and major depressive disorder. The Stanley Neuropathology Consortium. Mol. Psychiatry.

[45] Hayes S. (1994). Acetazolamide in Bipolar Affective Disorders. Ann. Clin. Psychiatry.

[46] Garvin P., Nilsson L., Carstensen J., Jonasson L., Kristenson M. (2008). Circulating Matrix Metalloproteinase-9 Is Associated with Cardiovascular Risk Factors in a Middle-Aged Normal Population. PLoS ONE.

[47] Garvin P., Nilsson L., Carstensen J., Jonasson L., Kristenson M. (2009). Plasma Levels of Matrix Metalloproteinase-9 are Independently Associated With Psychosocial Factors in a Middle-Aged Normal Population. Psychosom. Med..

[48] Rasic I., Rebic V., Rasic A., Aksamija G., Radovic S. (2018). The Association of Simultaneous Increase in Interleukin-6, C Reactive Protein, and Matrix Metalloproteinase-9 Serum Levels with Increasing Stages of Colorectal Cancer. J. Oncol..

